# Supplementary material for: Global warming reduces the carrying capacity of the tallest angiosperm species (Eucalyptus regnans)
Source: Nat Commun. 2025 Aug 21;16:7440. doi: 10.1038/s41467-025-62535-x (PMC12370954; doi:10.1038/s41467-025-62535-x)
Supplement: Supplementary file 1 — Supplementary Information [file 41467_2025_62535_MOESM1_ESM.pdf]

Supplementary Information: Global warming reduces the carrying capacity of the tallest angiosperm species (*Eucalyptus regnans*)

Raphaël Trouvé[1*], Patrick J. Baker[1], Mark J. Ducey[2], Andrew P. Robinson[3], and Craig R. Nitschke[1]

[1]The University of Melbourne, School of Agriculture, Food and Ecosystem Sciences, Richmond, Victoria, 3121, Australia.
[2]University of New Hampshire, Department of Natural Resources and the Environment, 114 James Hall, Durham, NH, 03824, USA.
[3]The University of Melbourne, CEBRA & School of BioSciences, Parkville, Victoria, 3010, Australia.
[*]Address correspondence to Raphaël Trouvé; raphael.trouve@unimelb.edu.au

Contents

Supplementary Note 1: Permanent sample plot database and sampling design . . 5
Supplementary Note 2: Impact of climate conditions on the static self-thinning allometry 7
Supplementary Note 3: Goodness-of-fits of the self-thinning models 10
Supplementary Note 4: Relative size of trees that died during a mortality event . 12

List of Figures

Supplementary Figure 1 Map of plot location 7
Supplementary Figure 2 Relationship between 30-year climate averages (1981-2010) of mean annual vapor pressure deficit and mean annual temperature 8

List of Tables

Supplementary Table 1 Summary statistics of the data 6
Supplementary Table 2 Parameter summary for the static self-thinning allometry models involving climate predictors. 9
Supplementary Table 3 Model goodness-of-fit, evaluated using leave-one-plot-out cross-validation. 11
Supplementary Table 4 Parameter summary for the linear mixed model of the relative size of trees that died (k factor) as a function of climate predictors. 13

Supplementary Note 1: Permanent sample plot database and sampling design

The study analysed even-aged stands of mountain ash (*Eucalyptus regnans*) in the Central Highlands of Victoria, Australia. The permanent sample plots come from a network of silvicultural experiments that were established in the mid-20th century by the Victorian Forestry Commission to study the effect of thinning on growth and yield. Each experimental site had several permanent plots and included a range of thinning treatments, plus unthinned control plots. Mean plot size was 2600 m^2 (ranging from 400 m^2 to 8000 m^2). Trees in each plot were measured for diameter at breast height (DBH, measured at 1.3 m height) and recorded as either live or dead every 2–3 years, with data collection continuing into the late 1990s or early 2000s. The data was originally collected by the Forestry Commission. When the Forestry Commission was dis-established in 1983, the Victorian Department of Energy, Environment, and Climate Action (DEECA) and its precursors managed and curated the data.

We selected pure *E. regnans* plots from the database based on the following criteria (1): more than 80% of the basal area was from *E. regnans*, plots were sufficiently large ($\geq$400 m^2) with sufficiently long intercensus intervals ($\geq$0.5 year) and growth ($\Delta D \geq 0.1$ cm year^{-1}). We excluded plots that experienced fire, psyllid infestation, or storm damage. For each plot and each measurement, we calculated the quadratic mean diameter (D in cm), tree density (trees ha^{-1}), and dominant height (Ho in m, defined as the height of the 100 largest trees per hectare). For each pair of successive measurements on the same plot, we counted the number of dead trees (ΔN, in trees) and calculated the net increase in quadratic diameter (ΔD, in cm) during that period. In total, we had 1302 measurements from 112 plots, including 328 measurements from 40 unthinned control plots that showed no evidence of having experienced disturbance over their measurement period (1). We used the control plots to calibrate static self-thinning allometries (Eq. 1 in manuscript) and all the plots to calibrate the mortality models (Eqs. 2 and 3 in the manuscript) which are used to derive survival trajectories that converge to the self-thinning line (1). The plot location is shown in Fig. 1 and the data is summarized in Table 1.

Supplementary Table 1: Summary statistics of the data

Data	Metrics	Count	Mean	Min	Max
All plots (mortality models)	n_{obs} (count)	1302			
	n_{plots} (count)	112			
	Measurement year		1976	1947	2000
	Age (years)		39.4	3.0	81.0
	Ho (m)		46.1	10.3	73.4
	N (stem ha^{-1})		540	44	39288
	D (cm)		46.6	2.8	110.7
	BA (m^2 ha^{-1})		42.3	1.0	98.7
	D increment (cm $year^{-1}$)		1.0	0.2	3.6
	Mortality rate (% $year^{-1}$)		1.6	0.0	41.4
	MAT (°C)		11.4	8.7	13.7
	P (mm $year^{-1}$)		1450	657	2092
	AHMI		15.4	9.4	35.1
	VPD (hPa)		10.1	9.3	11.4
Control plots (static allometry)	n_{obs} (count)	328			
	n_{plots} (count)	40			
	Measurement year		1977	1947	1997
	Age (years)		43.4	4.0	78.0
	Ho (m)		46.8	19.4	72.7
	N (stem ha^{-1})		965	156	39288
	D (cm)		42.4	2.8	76.3
	BA (m^2 ha^{-1})		61.5	24.6	98.7
	D increment (cm $year^{-1}$)		0.8	0.2	2.5
	Mortality rate (% $year^{-1}$)		4.7	0.0	41.4
	MAT (°C)		11.1	8.7	13.0
	P (mm $year^{-1}$)		1484	672	2008
	AHMI		14.8	9.8	31.6
	VPD (hPa)		9.9	9.3	11.2

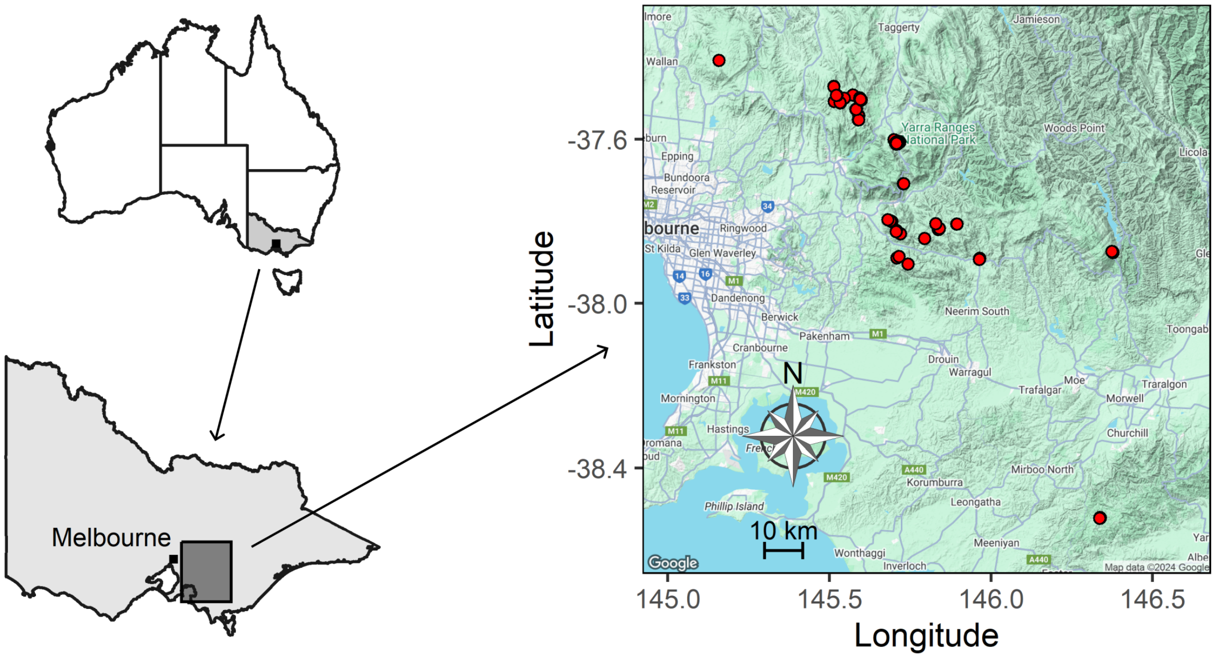

Supplementary Figure 1: Map of plot location in the Central Highlands of Victoria, to the east of Melbourne, Australia. Base map: Google Maps: 'Map data © 2024 Google'.

Supplementary Note 2: Impact of climate conditions on the static self-thinning allometry

In this section, we estimate and compare the impact of mean annual temperature (MAT), annual precipitation (P), annual heat moisture index (AHMI), and mean annual vapor pressure deficit (VPD) on the static self-thinning allometry. In the manuscript, only the best model (MAT) is presented.

The self-thinning line describes the maximum number of trees of a given mean size that can be stocked per unit area. Since the maximum stocking level described by the self-thinning line depends on the amount of resources available, we expect it to decrease as climatic conditions become warmer and drier.

We modelled the $\log(N)$ vs. $\log(D)$ relationship using a hierarchical linear model with a plot random effect on the intercept and tested for an effect of climatic conditions on the self-thinning line. We fitted three alternative models including either $\mathrm{MAT}_{1960-2000_j}$, $\mathrm{P}_{1960-2000_j}$, $\mathrm{AHMI}_{1960-2000_j}$, or mean annual $\mathrm{VPD}_{1980-2010_j}$. These variables are mean values per plot over the $1960-2000$ period, except for VPD which was averaged over 1981–2010 as earlier data were not available (2). We used a plot-level random effect to account for the fact that measurement from the same plots are not independent. The log-log linear mixed-effects model is as follows:

$$\begin{aligned} \log(N_{\mathrm{ij}}) &= \alpha_{0\mathrm{j}} + \alpha_1 \log(D_{\mathrm{i}}) + \alpha_2 \mathrm{Predictor}_j + \epsilon_{\mathrm{i}} \\ \alpha_{0\mathrm{j}} &\sim \mathcal{N}(\alpha_0, \sigma_{\alpha_0}) \\ \epsilon_{\mathrm{i}} &\sim \mathcal{N}(0, \sigma) \end{aligned} \tag{S1}$$

where i represents observation i in plot j. N is the stand density (number of trees per ha), D is the quadratic mean diameter, and $\mathrm{Predictor}_{\mathrm{j}}$ represents the 40-year (1961-

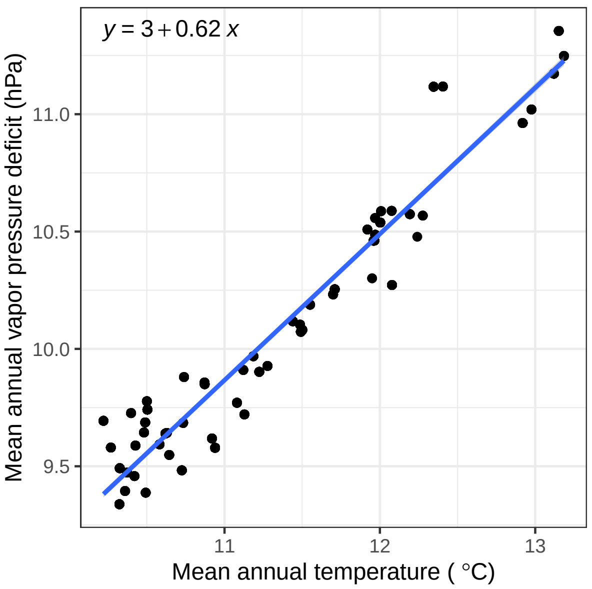

Supplementary Figure 2: Relationship between 30-year climate averages (1981-2010) of mean annual vapor pressure deficit and mean annual temperature across all plots. The solid line shows the linear regression fit (R = 0.96, p < 0.001).

2000) average of various climatic predictors for the plot (*i.e.*, MAT, precipitations, and AHMI). α_{0j} is the intercept of the self-thinning line for plot j, α_1 is the slope of the self-thinning line, and $alpha_2$ represents the potential impact of various climate predictors on the intercept of the self-thinning line[1]. σ_{α_0} is the standard deviation for the plot-level random effect and σ is standard deviation of the residuals. We used weakly informative priors for model fitting (3). Parameters estimates for Eq. S1 are shown in Table 2.

Out of the four climatic predictors tested, both mean annual temperature (MAT) and vapor pressure deficit (VPD) significantly affected the self-thinning line (Table 2). Using a leave-one-plot-out cross-validation procedure, the MAT model showed slightly better goodness-of-fit than the VPD model (see section 3). Given this result and the strong correlation between MAT and VPD in our plots (R = 0.96, p < 0.001, see Fig. 2), along with practical constraints (VPD data only extends back to 1981 versus 1947 for MAT and lacks reliable climate change projections), we focused our subsequent analyses on the MAT model. Regarding the impact of MAT, we found that for each degree increase in temperature among plots, the intercept of the self-thinning line decreases by 0.09, representing a 9% decrease in carrying capacity (number of stems per hectare for a stand of a given size).

[1]Note that climate predictors were centred to have a mean of zero by subtracting their average value from the dataset. For example, we used ($MAT_{1961\text{-}2000j}$ - 11.48) instead of $MAT_{1961\text{-}2000j}$ as predictor. This means that α_0 represents the intercept of the self-thinning line under average climate conditions. Precipitation and AHMI were further scaled to have a standard deviation of one so that the predictors on a similar scale. The $MAT_{1961\text{-}2000j}$ and VPD predictors were not scaled because: 1) this would reduce the interpretability of the α_2 parameter which can currently be interpreted as the impact of a one degree increase in temperature on the self-thinning intercept; and 2) the standard deviation for the $MAT_{1961\text{-}2000j}$ predictor is 0.89, which was deemed sufficiently close to one to allow a comparison with the scaled precipitation and AHMI predictors which have a standard deviation of one.

Supplementary Table 2: Parameter summary for the static self-thinning allometry models involving climate predictors. Note that we subtracted the mean of climate predictors values so that they have a mean of zero (α_0 thus represents the intercept of the self-thinning line for mean climatic conditions found in the dataset). Precipitation and AHMI were further standardised to have a standard deviation of one.

			95% CI	
Parameter	Predictor	Mean	Min	Max
α_0	Intercept	12.29	12.18	12.40
α_1	log(D$_i$)	-1.67	-1.69	-1.64
α_2	centred $MAT_{1961-2000_j}$	-0.09	-0.14	-0.05
σ_{α_0}	Plot-level SD	0.12	0.10	0.16
σ	Residual SD	0.06	0.05	0.06
α_0	Intercept	12.29	12.18	12.40
α_1	log(D$_i$)	-1.66	-1.69	-1.63
α_2	scaled $P_{1961-2000_j}$	0.01	-0.03	0.05
σ_{α_0}	Plot-level SD	0.15	0.12	0.19
σ	Residual SD	0.06	0.05	0.06
α_0	Intercept	12.30	12.18	12.40
α_1	log(D$_i$)	-1.66	-1.69	-1.63
α_2	scaled $AHMI_{1961-2000_j}$	-0.01	-0.03	0.01
σ_{α_0}	Plot-level SD	0.14	0.11	0.18
σ	Residual SD	0.06	0.05	0.06
α_0	Intercept	12.30	12.18	12.39
α_1	log(D$_i$)	-1.67	-1.69	-1.64
α_2	centred $VPD_{1981-2010_j}$	-0.12	-0.19	-0.04
σ_{α_0}	Plot-level SD	0.13	0.10	0.16
σ	Residual SD	0.06	0.05	0.06

Supplementary Note 3: Goodness-of-fits of the self-thinning models

This section describes the goodness of fit for the static self-thinning allometry and mortality models, estimated using a leave-one-plot-out cross-validation procedure. We tried several climate variables. For the static self-thinning allometry (Eq. 1 in the manuscript), we tested $\mathrm{MAT}_{1961-2000_j}$, $\mathrm{Precipitation}_{\mathrm{1961\text{-}2000j}}$, and $\mathrm{AHMI}_{\mathrm{1961\text{-}2000j}}$, as well as a model without climate variables. For the mortality model (Eqs. 2 and 3 in the manuscript), we tested $\mathrm{MAT}_{\mathrm{1961\text{-}2000j}} + \mathrm{MAT}_{\mathrm{anomaly_i}}$ and $\mathrm{MAT_i}$ as well as a model without climate variables. We did not test precipitation and AHMI predictors in the mortality model because they were not significant in the static self-thinning allometry model screening.

In leave-one-plot-out cross-validation, the model is trained using data from all but one plot, with the remaining plot used to test the model's predictive performance in out-of-sample data. This process is repeated for every plot, where each time, a different plot is left out for testing, and the model is re-calibrated on the remaining plots. After each iteration, the model's predictions for the held-out plot are saved. At the end of the process, the predictions for all plots are combined to evaluate the model's out-of-sample predictions against observations.

We then calculated the coefficient of determination (R^2), the root mean square error (RMSE), and the bias for each model using the out-of-sample data. The models were evaluated based on their ability to predict $\log(N)$ for the static allometry models and mortality rates for the mortality models. The goodness-of-fits metrics follow:

$$\mathrm{R}^2 = 1 - \frac{\sum(y_\mathrm{i} - \widehat{y}_\mathrm{i})^2}{\sum(y_\mathrm{i} - \overline{y})^2} \tag{S2}$$

$$\mathrm{RMSE} = \sqrt{\frac{\sum(y_\mathrm{i} - \widehat{y}_\mathrm{i})^2}{n}} \tag{S3}$$

$$\mathrm{BIAS} = \frac{\sum y_\mathrm{i} - \widehat{y}_\mathrm{i}}{n} \tag{S4}$$

where y_i is the observed value (*i.e.*, $\log(N)$ for the static self-thinning allometry models and mortality count and mortality rates for the the mortality models) for inventory i and $\widehat{y}_\mathrm{i}$ is the model's prediction. $\overline{y}$ is the average observed value in the dataset.

Observed mortality rates were calculated based on successive measurements on the same plot using the following equation:

$$\frac{\log(N_{\mathrm{t1}}) - \log(N_{\mathrm{t2}})}{t2 - t1} \tag{S5}$$

where N_{t1} is tree density per ha at time $t1$ and N_{t2} is tree density per ha at time $t2$.

To compute predicted mortality rates, we used an algebraic equation predicting N_{t2} based on N_{t1}, D_{t1}, D_{t2}, and climatic variable inputs. The algebraic equation is derived by integrating the mortality equation from size D_{t1} to size D_{t2} (see 1, for a derivation). The algebraic equation was chosen over a naive estimate of mortality count because it better matches how survival trajectories are projected in practice (1) and it also makes it easier to adjust the projection length. The algebraic equation from **(author?)** (Eq.5 in 1) is reproduced below for convenience:

$$N_{t2} = \left(N_{t1}^{-\beta_2} + exp(\beta_0)\frac{-\beta_2}{\beta_1+1}(D_{t1}^{\beta_1+1} - D_{t2}^{\beta_1+1})\right)^{\frac{1}{-\beta_2}} \tag{S6}$$

After predicting N_{t2} using Eq. S6, we use Eq. S5 to calculate the mortality rate predicted from our mortality model.

The goodness-of-fit of the models is shown in Table 3. The best model for the static self-thinning allometry was the model with $MAT_{1961\text{-}2000j}$. The best model for the mortality model was the model with MAT_i. Both model predict a 9% decrease in the intercept of the self-thinning line (*i.e.*, forest carrying capacity) for each additional °C increase in MAT.

Supplementary Table 3: Model goodness-of-fit, evaluated using leave-one-plot-out cross-validation. The performance of the models is measured by their ability to predict log(N) in static allometry models and mortality count and mortality rates in mortality models

Model	Climate predictor	R^2	RMSE	BIAS
Static allometries				
Response = log(N)	none	0.963	0.151	-0.017
	$MAT_{1961-2000_j}$	0.973	0.131	-0.010
	$P_{1961-2000_j}$	0.962	0.154	-0.021
	$AHMI_{1961-2000_j}$	0.963	0.151	-0.017
	$VPD_{1981-2010_j}$	0.970	0.136	-0.007
Mortality models				
Mortality count (tree ha^{-1} $year^{-1}$)	none	0.982	61.1	-5.2
	$MAT_{1961-2000_j} + MAT_{anomaly_i}$	0.984	58.5	-4.1
	MAT_i	0.984	58.9	-4.5
Mortality rate (% $year^{-1}$)	none	0.703	0.0196	-0.0051
	$MAT_{1961-2000_j} + MAT_{anomaly_i}$	0.717	0.0191	-0.0049
	MAT_i	0.719	0.0190	-0.0049

Supplementary Note 4: Relative size of trees that died during a mortality event

In this section, we report on the analysis of the relative size of trees that died during a mortality event. The database used for the relative dead tree size analysis is the same as the one used for the self-thinning analysis. The database originally contained 1302 plot measurements, including 601 where at least one tree in the plot was dead by the end of the period. In each of these cases, we recorded the quadratic mean diameter of the live trees at the beginning of the period and the quadratic mean diameter of the trees that died by the end. The ratio of these two measurements is often referred to as the k factor, where $k = \frac{D_{\text{dead}}}{D}$. To increase the signal-to-noise ratio of our analysis, we considered k factors where at least four trees died in the mortality event. We identified 273 such events, involving a total of 4728 dead trees.

The average k factor in our dataset was 0.62. This indicates that trees that died by the end of the measurement period were, on average, 38% smaller than the live trees at the beginning of the measurement period: dead trees tend to be smaller and suppressed compared to their neighbors.

We used linear mixed models to test whether the k factor was influenced by climatic factors (mean annual temperature, precipitation, AHMI), whether there was a trend over time (year), and stand dominant height (Ho). We included dominant height as taller trees might be more susceptible to drought due to increased hydraulic constraints (4). We used a plot-level random effect to account for the fact that measurement from the same plots are not independent. The models follow:

$$\begin{aligned} k_{\text{ij}} &= \gamma_{0\text{j}} + \gamma_1 \text{Predictor}_{\text{i}} + \epsilon_{\text{i}} \\ \gamma_{0\text{j}} &\sim \mathcal{N}(\gamma_0, \sigma_{\gamma_0}) \\ \epsilon_{\text{i}} &\sim \mathcal{N}(0, \sigma) \end{aligned} \tag{S7}$$

where k_{ij} represents the k factor observed for observation i in plot j. γ_{0j} is the intercept of the regression for plot j and γ_1 represents the potential impact of various predictors (*i.e.*, mean annual temperature, precipitation, and AHMI, and year) on the k factor[2]. σ_{γ_0} is the standard deviation for the plot-level random effect and σ is standard deviation of the residuals. We used weakly informative priors for model fitting (3). Parameters estimates for Eq. S7 are shown in Table 4.

The analysis confirmed that trees found dead at the end of the measurement period were, on average, 0.62 to 0.64 times smaller than the mean tree size at the beginning of the period (see intercept estimates in Table 4). None of the tested predictors – climate variables, time trends, or dominant height – significantly affected the k factor (Table 4),

[2] Note that the climate predictors and observation years were centred to have a mean of zero by subtracting their average value from the dataset. For example, we used (MAT_{i} - 11.43) instead of MAT_{i} as predictor. This means that γ_0 represents the mean k factor under average climate conditions. Precipitation, AHMI, Year, and Ho predictors were further scaled to have a standard deviation of one so that all predictors on a similar scale. The MAT_{i} predictor was not scaled because: 1) this would reduce the interpretability of the γ_1 parameter which can currently be interpreted as the impact of a one degree increase in temperature on the k factor; and 2) the standard deviation for the MAT_{i} predictor is 0.94, which we deemed sufficiently close to one to allow a comparison with the scaled precipitation, AHMI, Year, and Ho predictors which have a standard deviation of one.

Supplementary Table 4: Parameter summary for the linear mixed model of the relative size of trees that died (k factor) as a function of climate predictors.

			95% CI	
Parameter	Predictor	Mean	Min	Max
γ_0	Intercept	0.64	0.62	0.67
γ_1	centred $\mathrm{MAT_i}$	-0.01	-0.03	0.02
σ_{γ_0}	Plot-level SD	0.08	0.05	0.10
σ	Residual SD	0.08	0.08	0.09
γ_0	Intercept	0.64	0.62	0.67
γ_1	scaled $\mathrm{P_i}$	0.01	-0.01	0.02
σ_{γ_0}	Plot-level SD	0.07	0.05	0.10
σ	Residual SD	0.08	0.08	0.09
γ_0	Intercept	0.64	0.62	0.66
γ_1	scaled $\mathrm{AHMI_i}$	-0.01	-0.02	0.01
σ_{γ_0}	Plot-level SD	0.08	0.05	0.10
σ	Residual SD	0.08	0.08	0.09
γ_0	Intercept	0.64	0.62	0.66
γ_1	scaled $\mathrm{Year_i}$	0.00	-0.02	0.01
σ_{γ_0}	Plot-level SD	0.07	0.05	0.10
σ	Residual SD	0.08	0.08	0.09
γ_0	Intercept	0.62	0.60	0.65
γ_1	scaled $\mathrm{Ho_i}$	0.00	-0.03	0.02
σ_{γ_0}	Plot-level SD	0.06	0.04	0.08
σ	Residual SD	0.09	0.08	0.10

indicating that suppressed trees were consistently more likely to die than other trees across all conditions.

References

[1] Trouvé, R., Nitschke, C. R., Robinson, A. P. & Baker, P. J. Estimating the self-thinning line from mortality data. *Forest Ecology and Management* **402**, 122–134 (2017). URL http://www.sciencedirect.com/science/article/pii/S0378112717308320.

[2] Stewart, S. B. & Nitschke, C. R. Improving temperature interpolation using modis lst and local topography: a comparison of methods in south east australia. *International Journal of Climatology* **37**, 3098–3110 (2017). URL https://rmets.onlinelibrary.wiley.com/doi/abs/10.1002/joc.4902.

[3] Gelman, A. *et al. Bayesian Data Analysis. Third edition* (Chapman and Hall, 2014).

[4] Ryan, M. G. & Yoder, B. J. Hydraulic limits to tree height and tree growth. *Bioscience* **47**, 235–242 (1997). URL https://doi.org/10.2307/1313077.
