# Supplementary material for: Global warming reduces the carrying capacity of the tallest angiosperm species (Eucalyptus regnans)
Source: Nat Commun. 2025 Aug 21;16:7440. doi: 10.1038/s41467-025-62535-x (PMC12370954; doi:10.1038/s41467-025-62535-x)
Supplement: Supplementary file 2 — Reporting Summary [file 41467_2025_62535_MOESM2_ESM.pdf]

nature portfolio

Corresponding author(s): Raphael Trouve

Last updated by author(s): Jun 18, 2025

Reporting Summary

Nature Portfolio wishes to improve the reproducibility of the work that we publish. This form provides structure for consistency and transparency in reporting. For further information on Nature Portfolio policies, see our Editorial Policies and the Editorial Policy Checklist.

Statistics

For all statistical analyses, confirm that the following items are present in the figure legend, table legend, main text, or Methods section.

n/a	Confirmed	
☐	☒	The exact sample size (*n*) for each experimental group/condition, given as a discrete number and unit of measurement
☐	☒	A statement on whether measurements were taken from distinct samples or whether the same sample was measured repeatedly
☐	☒	The statistical test(s) used AND whether they are one- or two-sided<br>*Only common tests should be described solely by name; describe more complex techniques in the Methods section.*
☐	☒	A description of all covariates tested
☐	☒	A description of any assumptions or corrections, such as tests of normality and adjustment for multiple comparisons
☐	☒	A full description of the statistical parameters including central tendency (e.g. means) or other basic estimates (e.g. regression coefficient) AND variation (e.g. standard deviation) or associated estimates of uncertainty (e.g. confidence intervals)
☐	☒	For null hypothesis testing, the test statistic (e.g. *F*, *t*, *r*) with confidence intervals, effect sizes, degrees of freedom and *P* value noted<br>*Give P values as exact values whenever suitable.*
☒	☐	For Bayesian analysis, information on the choice of priors and Markov chain Monte Carlo settings
☐	☒	For hierarchical and complex designs, identification of the appropriate level for tests and full reporting of outcomes
☒	☐	Estimates of effect sizes (e.g. Cohen's *d*, Pearson's *r*), indicating how they were calculated

Our web collection on statistics for biologists contains articles on many of the points above.

Software and code

Policy information about availability of computer code

Data collection	The data used in this study are available in the Zenodo repository (https://doi.org/10.5281/zenodo.15686632)
Data analysis	We fitted all models using the `brms' package (version 2.20.4) in R (version 4.4.1.). The R code used in the study is available in the Zenodo repository (https://doi.org/10.5281/zenodo.15686632)

For manuscripts utilizing custom algorithms or software that are central to the research but not yet described in published literature, software must be made available to editors and reviewers. We strongly encourage code deposition in a community repository (e.g. GitHub). See the Nature Portfolio guidelines for submitting code & software for further information.

Data

Policy information about availability of data

All manuscripts must include a data availability statement. This statement should provide the following information, where applicable:

- Accession codes, unique identifiers, or web links for publicly available datasets
- A description of any restrictions on data availability
- For clinical datasets or third party data, please ensure that the statement adheres to our policy

The data used in this study are available in the Zenodo repository (https://doi.org/10.5281/zenodo.15686632).

Research involving human participants, their data, or biological material

Policy information about studies with human participants or human data. See also policy information about sex, gender (identity/presentation), and sexual orientation and race, ethnicity and racism.

Reporting on sex and gender	NA
Reporting on race, ethnicity, or other socially relevant groupings	NA
Population characteristics	NA
Recruitment	NA
Ethics oversight	NA

Note that full information on the approval of the study protocol must also be provided in the manuscript.

Field-specific reporting

Please select the one below that is the best fit for your research. If you are not sure, read the appropriate sections before making your selection.

☐ Life sciences ☐ Behavioural & social sciences ☒ Ecological, evolutionary & environmental sciences

For a reference copy of the document with all sections, see nature.com/documents/nr-reporting-summary-flat.pdf

Ecological, evolutionary & environmental sciences study design

All studies must disclose on these points even when the disclosure is negative.

Study description	Our research quantifies the impact of rising temperatures on Eucalyptus regnans, the tallest flowering plant on Earth, its carrying capacity (i.e., self-thinning line), and its ability to store carbon. Using a unique dataset from a permanent forest sample plot network spanning over five decades (1947–2000) in southeastern Australia, we found that warming increased mortality rates and reduced forest carrying capacity in this ecosystem. Detailed data descriptions can be found in the Appendix. The raw data and analysis code are available in the supplementary materials. We found a projected 3°C rise by 2080 could decrease tree density and carbon stocks in these forests by 24%. We discuss the implications for forest conservation and management.
Research sample	A group of tagged Eucalyptus regnans trees located in plots distributed throughout the forested landscape. The population represents the tall, open wet forests (mountain ash forests) of southeastern Australia. The data was originally collected by the Forestry Commission. When the Forestry Commission was disbanded in 1983, the Victorian Department of Energy, Environment, and Climate Action (DECCA) and its predecessors managed and curated the data.
Sampling strategy	We selected all forest plots present in the curated database. The permanent sample plot network was installed to represent productive timber forests in the region. We filtered for Eucalyptus regnans monoculture (>80% basal area of E. regnans) and disturbances (fire, psyllid) to focus on competition-related mortality (baseline forest dynamics) rather than disturbance events.
Data collection	Tree mensuration (diameter measurement and tree status of all trees > 10 cm DBH). Climate data were obtained from the Australian Bureau of Meteorology.
Timing and spatial scale	Data were collected from 1947 to 2000. Trees were remeasured on average every 2 to 3 years. The data covers most of the Central Highlands of Victoria, the primary region where Eucalyptus regnans is found in the State.
Data exclusions	Plots affected by fire or psyllid infestation were excluded from the dataset to focus solely on competition-related tree mortality, rather than disturbance-related mortality.
Reproducibility	We provided R code and data to reproduce the methods and analysis.
Randomization	NA
Blinding	NA

Did the study involve field work? ☒ Yes ☐ No

Field work, collection and transport

Field conditions	Long-term forest surveys

Location	Central Highlands of Victoria, Australia. A map of plot location is available in Supplementary Information, Fig. 1. Summary data description is available in Supplementary Table 1. The raw data used in this study are available in the Zenodo repository (https://doi.org/10.5281/zenodo.15686632).
Access & import/export	Tree measurement data is stored in a database managed by the Victorian government (the current institution is DEECA, Victoria, Australia). Climate raster data can be downloaded from the Australian Bureau of Meteorology. We provided the data and R code in a supplementary material folder accompanying the manuscript.
Disturbance	We filtered plots that were affected by fire and psyllids.

Reporting for specific materials, systems and methods

We require information from authors about some types of materials, experimental systems and methods used in many studies. Here, indicate whether each material, system or method listed is relevant to your study. If you are not sure if a list item applies to your research, read the appropriate section before selecting a response.

Materials & experimental systems

n/a	Involved in the study	
☒	☐	Antibodies
☒	☐	Eukaryotic cell lines
☒	☐	Palaeontology and archaeology
☒	☐	Animals and other organisms
☒	☐	Clinical data
☒	☐	Dual use research of concern
☐	☒	Plants

Methods

n/a	Involved in the study	
☒	☐	ChIP-seq
☒	☐	Flow cytometry
☒	☐	MRI-based neuroimaging

Plants

Seed stocks	NA
Novel plant genotypes	NA
Authentication	NA
